# From Surviving Cancer to Getting on With Life: Adult Testicular Germ Cell Tumor Survivors’ Perspectives on Transition From Follow-Up Care to Long-Term Survivorship

**DOI:** 10.1177/10497323231173808

**Published:** 2023-06-05

**Authors:** Sandrien Weda, Danielle Zweers, Britt B. M. Suelmann, Richard P. Meijer, Sigrid C. J. M. Vervoort

**Affiliations:** 1 Department of Nursing Studies, 8119 University of Applied Sciences Utrecht, Utrecht, The Netherlands; 2 Department of Medical Oncology, 8124 UMC Utrecht, Utrecht, The Netherlands; 3 Department Oncological Urology, 8124 UMC Utrecht, Utrecht, The Netherlands; 4 Department of Nursing Science at Julius Center for Health Science and Primary Care, 8124 UMC Utrecht, Utrecht, The Netherlands

**Keywords:** testicular neoplasms, testicular germ cell tumor, cancer survivorship, cancer survivors, AYA

## Abstract

With an increasing incidence and a high cure rate, a growing number of testicular germ cell tumor (TGCT) survivors require specialized follow-up care. However, knowledge of these patients’ needs is lacking, leaving TGCT survivors with unmet care needs at risk of symptom burden when transitioning to long-term survivorship. This grounded theory study aimed to understand the perspectives of TGCT survivors’ transition from follow-up care to long-term survivorship. A total of 12 adult TGCT survivors in follow-up care or completion less than a year were in-depth semi-structured interviewed. Interviews were audiotaped and transcribed verbatim. Transcripts were analyzed by constant comparison, and the core category *“Dealing with back-and-forth forces”* emerged in the integrated concepts. Two comparative processes in dealing with those forces were identified: the process of *Living beyond the sword of Damocles* involved the transition from feeling threatened by cancer to overcoming those threats; the process of *Getting on with one’s life* can be described as transitioning from a period where cancer overruled their lives to carrying on with everyday life. The processes toward long-term survivorship follow general characteristics; the transition itself is an individual journey that depends on (life) experiences. The constructed model can guide healthcare professionals and researchers involved in TGCT survivorship to understand TGCT survivors’ individual and ensuing needs. When TGCT survivors receive individualized and tailored follow-up care, it can assist in preventing and reducing long-term and late effects on long-term survivorship.

## Background

Given the relatively young age at diagnosis and a 5-year survival rate exceeding 95%, depending on prognostic-based staging and pathological risk factors, a growing number of testicular germ cell tumor (TGCT) survivors require follow-up care after cancer treatment (Park et al., 2018; SEER, (n.d.); Trama et al., 2015). Testicular germ cell tumors (TGCTs) predominantly affect most men between 18 and 40 years of age, also known as Adolescents and Young Adults (AYA) (Cheng et al., 2018). When diagnosed early, TGCTs are one of the most treatable cancers, even in the case of metastases (Park et al., 2018). Despite the good prognosis, cancer and its treatments can result in adverse and late effects that significantly impact TGCT survivors’ lives. Existing studies concerning TGCT survivors’ lives have been conducted on the physical, social, psychological, and existential impact of adverse and late events of cancer and its treatment (Chovanec et al., 2021; Haugnes et al., 2012; Oechsle et al., 2016; Smith et al., 2013, 2018). Adverse events are defined as long-term effects and late events as effects that manifest months to years after cancer treatment (Aziz, 2007). For example, TGCT survivors can suffer from sexual problems including sexual dysfunction peripheral neuropathy, hearing loss and cognitive; and psychosocial problems including fear of cancer recurrence (Eberhard et al., 2009; Fung et al., 2018; Honecker et al., 2018; Pedersen et al., 2012; Rossen et al., 2012; Skaali et al., 2009). The results of the studies about the impact of adverse and late events on quality of life are ambiguous (Dahl et al., 2005; Fleer et al., 2004; Mykletun et al., 2005; Rossen et al., 2009). More recent studies have shown deterioration in specific QoL dimensions for a subset of TGCT survivors who experience sexual impairments or anxiety in the long term (Schepisi et al., 2019; Soon et al., 2019).

Follow-up care consists mainly of medical check-ups to monitor cancer recurrence and identify adverse and late events (Albers et al., 2019; Honecker et al., 2018). In addition, it aims to prepare TGCT patients for life after follow-up care. Understanding how TGCT survivors find a “new normal,” primarily related to the (self-)management of adverse and late events of cancer and its treatment, is desired to help them let go of follow-up care. Transforming into the “new normal” means becoming—and being seen as—a long-term cancer survivor (Mullan, 1985). Long-term survivorship is defined as survivors who are alive without evidence of disease for 5 years or more after their initial diagnosis. However, little attention has been paid to how the cancer experience pauses one’s life, particularly regarding TGCT perspectives on finding a “new normal” after cancer treatment.

Like any illness, having (had) cancer can be described as a transitional period in which survivors change from one stage to another in their journey of recovery and survivorship. One study described young adult cancer survivors' positive transformation after cancer treatment, not only for themselves but also for their close family members (Head & Iannarino, 2019). Although most cancer patients go through similar stages of survival, the meaning of the end of therapy and shifts in physical, social, psychological, and existential well-being toward long-term survivorship has been little examined. Two studies explored changes in the sense of self directly after cancer treatment and found standard and population-specific views in cancer survivors (Brodsky, 1995; von Werthern et al., 2022). As such, the transition in this study is examined as coping with a new situation rather than the vagaries that patients must overcome after cancer treatment. Although the studies mentioned above highlighted the experienced transformation of young adult cancer patient after active cancer treatment, little is known about population-specific changes in the transition to long-term survivorship. One study suggests that some testicular cancer patients struggle in the transition from cancer treatment to follow-up care, affecting their long-term health, and may benefit from additional information and support (Shen et al., 2016). These struggles were also seen from a clinical point of view, as TGCT survivors have difficulties ending their hospital visits and wish to continue follow-up care. No studies have looked specifically at the transition process to long-term survivorship. In other words, the exploration of how TGCT survivors transition from follow-up care to long-term survivorship and how they deal with this process is limited. This study aimed to explore testicular cancer survivors’ perspectives and how they construct their realities through their lived experiences on their journey to long-term survivorship. The study aimed to understand better the underlying process and what it means to transition. As a consequence, healthcare professionals can provide tailored care to TGCT survivors in preparation for life after follow-up care.

## Theoretical Framework

The study utilized the theory of locus of control. Because overcoming a life-threatening disease and dealing with the aftereffects of cancer and its treatment can challenge a person’s coping, it is necessary to examine factors that influence the response to a new situation. The transition process from follow-up care into long-term survivorship can challenge coping as it comes with many internal and external stressors (e.g., fear of recurrence and adjustments in work or education). The locus of control is relevant to TGCT survivors’ experiences because it enlightens the individual’s belief of the control they have over their life courses (internal locus of control) versus the control that external influences have over their lives (external locus of control) (Rotter, 1966). Some studies showed that an internal health locus of control positively affects well-being in cancer survivorship and that a loss of coping competence is related to the cancer experience (Hodges & Winstanley, 2012; L'Hotta et al., 2022). From a clinical point of view, this assumption supports the idea that TGCT survivors with an external locus of control need structural support more than TGCT survivors with an internal locus of control. How survivors respond to their locus of control can affect their transition process and, consequently, their needs during this process.

## Methods

### Study Design

Grounded theory methodology was chosen to explore TGCT survivors’ perspectives on the transition toward long-term survivorship and construct a model grounded in and derived from the data (Corbin & Strauss, 2015). The approach of this qualitative design fits the limited explored study area and assists in understanding the phenomenon. An iterative data gathering and analysis process was used to develop a theoretical explanation of the transition process. To understand the survivors’ lived experiences and assemble subjective evidence, we tried to get as close as possible to the studied survivors. Thick data have been gathered to unfold comparative processes in the TGCT survivors’ descriptions and address the diversity in the present population.

### Sample and Recruitment

TGCT survivors were first purposefully selected based on their experiences in the transition from hospital follow-up care to long-term survivorship. Survivors were eligible to participate if they were adults (≥ 18 years), were treated for (recurrence of) TGCT regardless of the type of germ cell tumor, were in complete remission, and were in follow-up care or completed follow-up care less than 1 year. Survivors were not eligible if they could not read and speak Dutch or had a mental or cognitive impairment. A diverse sample was obtained to provide the broadest range of individual perspectives on the transition from follow-up care into long-term survivorship. Survivors were purposefully selected with regard to variation in the stage in which they were in relation to the transition (before, during, and after the transition into long-term survivorship), age, cancer treatment, follow-up duration, educational background, and social status. Following an inductive-iterative approach, theoretical sampling was used until theoretical saturation was obtained (Corbin & Strauss, 2015). During the analytical process, new participants were selected based on their contribution to further developing the emerging theory concepts. For example, participants were sampled from survivors who had discussed their struggle with finishing follow-up care during a consultation and those who did not. Survivors who failed to transition into long-term survivorship and continued their follow-up care in the hospital, including medical check-ups, were sampled as deviant cases. These cases verified and broadened the concepts. The research team frequently discussed during the analytical process which characteristics of new participants could help to support the emerging theory.

TGCT survivors were recruited from an academic oncology outpatient clinic in the Netherlands. Eligible survivors were asked to participate during follow-up consultation by the clinical nurse practitioner (DZ). Those who expressed interest received an e-mail with study information from the researcher (SW). If the patient agreed, the researcher telephoned the patient within 2 weeks to give further information. If the patient was willing to participate, an appointment was made. Non-responding patients received a voicemail and were contacted again within 1 week to elevate response. Of the 16 eligible participants identified, 12 agreed to participate. Two non-participants declined, and two did not respond to the voicemail.

### Data Collection

Twelve semi-structured interviews were conducted via face-to-face or video calls using an interview guide (Table 1). Initially, face-to-face interviewing was chosen for data collection. Due to the COVID-19 outbreak, the following interviews were web-based with end-to-end-encrypted video call software. Firstly, participants were interviewed at the outpatient clinic. Digitally interviewed participants received guidelines to optimize the interview setting at their homes. Informed consent was obtained before the interview started: written consent for the face-to-face interviews and audio-recorded consent for the video calls.

**Table 1.** Interview Guide With Initial Topic List and Prompts.

Topic heading	Prompts
Experiences of the follow-up care itself	How have you experienced follow-up care? Can you tell me more about these experiences? Can you explain in your own words what follow-up care entails?
Experiences and expectations toward the ending of follow-up care	What does it mean for you that the follow-up care is nearing its end? What do you think about the idea that you will no longer have follow-up care? What do you need to complete the follow-up care? *If the participant shows signs of anxiety about completing the follow-up care:* Why are you worried? Why do you find the completion difficult? How do you handle these worries? What will you do with these worries? *If the participant shows signs of readiness toward the completion of the follow-up care:* Why are you ready to complete the follow-up care?
(Expectations of) Life after follow-up care	What will you do after the completion of the follow-up care?

The interview guide contained a topic list based on the theoretical framework and was continuously reframed during the iterative process to refine and check the concepts in the emerging theory. The initial topics were based on literature and expert opinions of clinical professionals caring for TGCT survivors (Haugnes et al., 2012; Nathan et al., 2011; Smits-Seemann et al., 2017). All interviews started with the same question: “Can you describe what follow-up care means/has meant for you?”. To collect demographic characteristics, interviews ended with closed questions on age, cancer treatment, follow-up duration, education, and social status.

Interviews were conducted between February and June 2020 by a Master’s student in Health and Life Sciences (SW) trained in conducting interviews. The interviewer (SW) worked as a nurse at an academic oncology inpatient clinic in the Netherlands and was familiar with the field of TGCT. The interviewer was not known to the participants before the sample recruitment. All interviews were audio-recorded. Memos were made during and directly after interviews on ideas about the concepts in the emerging theory and the reframing of the interview guidelines. Data collection ended after theoretical saturation was reached and confirmed during the analysis of the last two interviews (Corbin & Strauss, 2015).

### Data Analysis

The data analysis was conducted by two researchers (SW and SV) and supported by NVivo software (v12.6, QRS International). Data were analyzed using constant comparison (Corbin & Strauss, 2015). Interviews were transcribed verbatim and anonymized by one researcher (SW). Firstly, to get familiar with the data, the transcripts were read out in full to get an overall view and re-read to grasp the details. Meaningful phrases were singled out line-by-line and in-vivo coded, leading to initial concepts and fragments. Thereafter, the fragmented data with similar traits were collated into categories. New data was compared with the constructed categories to develop and verify the emerging theory. The categories were further arranged as links between the categories were identified and structured into processes and themes (Corbin & Strauss, 2015). In this phase, the outline of the core category as the basis for the transition process was formed. A preliminary model was constructed and compared with the original transcripts. Data analysis in this stage consisted of constantly comparing the conceptual level of the collected data and exploring differences and similarities across the data. New insights within the concepts were combined with those that emerged previously. Data were coded and analyzed independently by two researchers (SW and SV), and throughout the entire research process discussed until consensus was reached. A third researcher (DZ) read the transcripts, checked the coding, and discussed her opinion if different, being able to verify the preliminary model and its processes and themes. At the end of this stage, the themes and core category were refined and the core category *“Dealing with back-and-forth forces”* was affirmed. The researchers (SW, DZ, and SV) selected the most prominent processes and themes, illustrating vivid quotes in consensus. The research team reviewed the main processes and their links and worked toward an agreement about the interpretations, and finally, the theoretical model was defined. During each phase, reflexive and methodological memos were generated to record the analysis process and analytical reflections.

### Ethics

The study was conducted according to the principles of the Declaration of Helsinki (version October 2013) and following Medical Research Involving Human Subjects Act (WMO). The Medical Research Ethics Committee (MREC) Utrecht confirmed that the Medical Research Involving Human Subjects Act (WMO) does not apply to the study and that therefore an official approval is not required (reference number WAG/mb/20/005583). Guidelines for conducting qualitative studies established by the Standards for Reporting Qualitative Research (SRQR) were adopted (O’Brien et al., 2014).

### Credibility

Corbin and Strauss (2015) determine the quality of qualitative research to the degree that the study reflects TGCT survivors’ life experiences and resonates with researchers’ and readers’ experiences, the grounded theory’s credibility. In this study, as Corbin and Strauss (2015) indicated, credibility is fostered by sensitivity to participants and the data, self-awareness, training in conducting qualitative research, and methodological consistency and awareness. The research team consisted of a diverse team of oncologists, an oncology nurse practitioner, a nurse in oncology, and a senior nursing researcher trained in qualitative research to follow investigator triangulation. Most members were experts in the field of TGCT and verified the results. The interviewer (SW) was trained in qualitative interviewing techniques, which were evaluated by the research team through a pilot interview. Vivid quotes were translated by a bilingual academic student in Dutch and English to stay close to the original data. We acknowledge the researcher’s role in the research process and how it can affect the data collection. The position and preconceptions of all involved researchers were given due consideration to their prior experience with and understanding of TGCT and follow-up care. For example, the clinical nurse practitioner (DZ) had no engagement with data generation and initial coding to ensure the separation of the patient and consultant perspectives. The interviewer (SW) was a nurse and Master’s student in Health and Life Sciences. The interviewer was able to empathize with the investigated phenomenon as the interviewer worked with TGCT cancer patients in an inpatient oncology clinic. To enable participants to speak freely about a positive and negative experience, the interviewer’s role was explained as not being part of the clinical team and would preserve confidentiality accordingly. From the beginning of the data collection and throughout the study, theoretical memos and an audit trail were logged by one researcher (SW) and shared and discussed with the other researchers (DZ and SV) to increase methodological consistency (Corbin & Strauss, 2015). The results were continually discussed to ensure consistency and coherence and that the analysis remained grounded in the data. Critical reflections to acknowledge their involvement in the study were part of these discussions to be aware of the personal thoughts and actions and how this could affect the study. Perspectives of medical professionals (BS and RM) in the field of TGCT were sought in the final stage of the data analysis. This reflective approach allowed us to, as far as possible, bracket off any preconceptions or subjective biases.

## Findings

### Demographic and Participant Characteristics

A total of twelve survivors were interviewed: ten survivors in follow-up care and two survivors who ended follow-up care (Table 2). Two survivors had experienced a recurrence of cancer. In all, one survivor was in the first year of follow-up care, two in their second year, three in their third year, two in their fourth year, and four in more than 5 years of follow-up care. The average age of survivors who participated in the interview was 33 years, ranging from 28 to 49 years. Most survivors worked full-time, one worked part-time, and one was unemployed. Half of the survivors had a vocational education and training background, and the other half had a higher educational background. Five survivors were married, four were in a relationship, two were single, and one was divorced. The cancer treatments varied from surgery alone; surgery and chemotherapy; surgery and radiotherapy; or surgery, chemotherapy, and radiotherapy.

**Table 2.** Demographic Data.

Characteristics	*N* (=12)
Age (in years)	
18–29	3
30–39	7
40–49	2
Educational background^a^	
Vocational education and training (VET)	6
Higher education	6
Employment	
Full-time	10
Part-time	1
Unemployed	1
Social status	
Married	5
In a relationship	4
Single	2
Divorced	1
Cancer treatment(s)	
Surgery alone^b^	2
Surgery and chemotherapy	8
Surgery and radiotherapy	1
Surgery, chemotherapy, and radiotherapy	1
Follow-up duration (in years)^c^	
0–1	1
1–2	2
2–3	3
4–5	2
>5	4

^a^Education system in the Netherlands.

^b^Surgery: orchiectomy and/or lymphadenectomy.

^c^Since the last start when having a cancer recurrence.

### Grounded Theory: Dealing With Back-and-Forth Forces Toward Long-Term Survivorship

**Figure 1.**
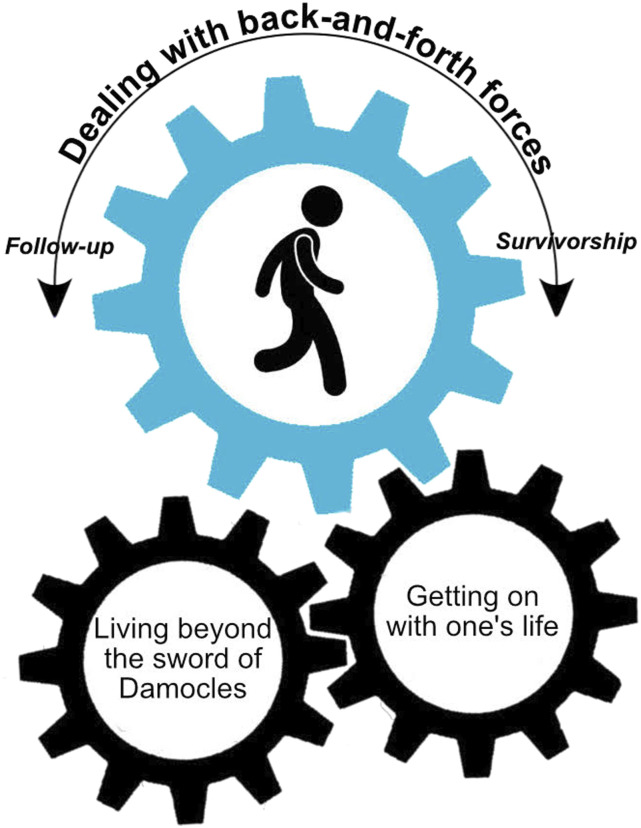
The transition model in cancer survivorship: Dealing with back-and-forth forces.

The central category “Dealing with back-and-forth forces” represents the core of TGCT survivors in coping with the transition from follow-up care to long-term survivorship. For TGCT survivors, the transition process involved dealing with threatening feelings of TGCT and what led to overcoming those threats to carry on with everyday life (Figure 1). The model shows a rotating movement in which the TGCT survivor walks in the grand cog toward long-term survival and influences how the processes in transition are moved with his steps.

The transition from follow-up care to long-term survivorship is conceptualized as an individual and cyclical journey involving two main processes: “*Living beyond the sword of Damocles”* and “*Getting on with one’s life”* (Figure 1). The phrase *“Living beyond the sword of Damocles”* was used by survivors to explain the catastrophic threat that is continuously looming over them. The process involved transitioning from feeling threatened by cancer to overcoming those threats. The process of “*Getting on with one’s life”* can be described as transitioning from a period where cancer overruled their life to carrying on with everyday life.

The individuality of the transition is represented by the grand cog in which the survivor rotates at their own pace from follow-up care to long-term survivorship. The speed and position of the processes can alternate due to individual (life) experiences. The model demonstrates that as the survivor moves forward in life and, therefore, moves away from follow-up care and hospital visits, he can get on with life. Cancer and all the medical checks are slowly taking a less prominent spot in the survivor’s life.

Then you can get on with your life. Then you can leave it all behind you ... from the start and then until my last check, then that process is completely finished. (P9)

As the feeling of living strengthens, it lessens the sense of impending doom. This is shown by the saying, “having a sword of Damocles hanging over their head,” meaning something terrible could happen imminently. In this context, it would mean the return of a cancer diagnosis. The model also works in the other direction. More follow-ups will increase the sense of looming doom and allow for less mental space to carry on with everyday life. The recurrence of cancer resets the grand cogwheel, restarting the transition. None of the participants moved at the same speed toward long-term survivorship, and different personal reasons were given for why they were able, or in some cases unable, to let go of follow-up care, even after 5 years of follow-up care. However, an overall pattern emerged, showing that all survivors were affected by the two main processes that set each other in motion. Each process involves three themes that can influence the transition (Table 3).

**Table 3.** Summary of the Processes and Themes in the Transition From Follow-Up Care to Long-Term Survivorship.

Process	Theme
Living beyond the sword of Damocles	Reaching a critical point to feel clean Finding reassurance in repeatedly good results Knowing that aid is always available if needed
Getting on with one’s life	Restoring daily routines Regaining confidence in one’s own body Mentally being able to move on

### Process: “Living Beyond the Sword of Damocles”

#### Theme: Reaching a critical Point to Feel Clean

Transitioning from being treated for cancer to living beyond cancer involved setting a critical point where the risk of cancer recurrence was below a certain percentile, known by low tumor markers, good imaging results, and oncological evaluations. Many participants marked this point as a sense of “*being clean*.”

Well, basically, I was clean when my tumor markers were at a normal level. (P9)

Survivors who had not reached this critical point yet described follow-up care as more intensive and stressful. Most participants who did overcome the critical point of a particular risk of cancer recurrence noticed that follow-up care was less intensive and time between consultations was more spread out. The change in intensity gave several participants a positive outlook on their recovery process. As time passed, most participants trusted more and more that they would stay clean.

As we are a year further since my last treatment, I can only see it as an assurance … especially since I have been clean for one and a half years. (P5)

Half of the survivors perceived decreasing appointments during follow-up care as slowly cutting the cord until they were declared medically cured of cancer at the final follow-up consultation.

Anyway, the goal is to heal and at a certain point, after years, the visits to the hospital wind down. I must say, it was part of it, and I believe this is all right. (P6)

#### Theme: Finding Reassurance in Repeated Good Results

At the start of follow-up care, all participants described more tension when waiting for tumor markers and imaging results. Receiving repeated good results meant for all participants less tension and growing trust in their bodies. Several participants described this reassurance as “*again knowing everything is all right*.” Letting go of the reassurance of good results was often a challenging part of the transition to long-term survivorship. This was clarified by the experience of participants with more than 5 years of follow-up care.

Trust has taken a hit, so if every half year it can be corrected, that is nice. (P3)

As the results stayed good, the participants noticed less involvement from their families and friends, which helped them release the need for reassurance. Some participants expressed that waiting for results also caused restlessness in their families and friends. The two participants who ended follow-up care requested a final checkup through imaging and tumor markers to ensure that nothing was missed during follow-up care.

All survivors described how cancer recurrence would remain an uncertainty in their lives. Nearly all participants spoke of the need to accept that sometimes fear of cancer recurrence would appear. A few survivors spoke of ways they learned to channel their fear of cancer recurrence.

I recognise the pattern, and I know how I should react to myself without getting myself completely worked up. (P5)

In more cases, survivors reached out for help from outside the hospital to develop tools for dealing with the fear of cancer recurrence.

…that when it reappears again, to be able to regulate it in a good way so that you will return to a normal sense as you normally would get from imaging and blood values. (P1)

#### Theme: Knowing that Aid Is Always Available if Needed

Participants felt that contacting their medical oncologist or clinical nurse about TGCT after finishing follow-up care made more sense than going to their general practitioner. The knowledge that men could always return to the outpatient clinic contributed to letting go of follow-up care and gave half of the participants a “*sense of security*.” For some survivors, and mainly those who were longer than 5 years in follow-up care, this reassurance did not help in letting go of their follow-up care:

… although I understand swimming, and I can swim, it is just a nice feeling that the lifeguard is still nearby. (P1)

### Process: “Getting on With One’s Life”

#### Theme: Restoring Daily Routines

Restoring daily routines and prioritizing them above follow-up care enabled men to get on with life. Through actively “*picking up the pieces*” and “*at their own pace*” restoring routines, men strived to get on with their daily lives. Most survivors spoke openly about their cancer histories with close ones to receive understanding. Some survivors sought professional help when restoring daily routines was perceived as problematic. Overall, men described that restoring their daily routines contributed to finding closure with cancer’s impact on their lives.

It was really hard for me, after all these checks, to pick up my studies or find a job…it is only after three years or so, I think, that emotionally I was ready to accept it all. I graduated, and I have a job. Everything, the whole picture. That enables me to give it all closure. (P7)

All participants noticed a shift in prioritizing follow-up care to daily life during follow-up care. Several participants perceived this as “*planning follow-up care around their daily life”* and not planning their lives around follow-up care. As time passed, a feeling of the routine was felt by certain participants (*“going there is now the same as going to the bakery*” (P6)). Some survivors said they sometimes even forgot their follow-up appointment (*“to be honest, I even have forgotten to do the checks”* (P3)). Survivors who were not open about their cancer history, which was more common in participants still in follow-up care after 5 years, experienced more difficulties restoring their daily routines and felt more alone and/or frustrated in handling the adverse and late effects of cancer treatment.

#### Theme: Regaining Confidence in One’s Own Body

The transition meant dealing with the aftereffects of cancer treatment as participants sought to re-establish trust in their bodies. For some participants, cancer felt like a betrayal by their body (*“Isn’t it weird that your body does this?”* (P6)). To trust their bodies again, they needed “*time*,” and their insecurities were treated in follow-up care. Some survivors who had learned self-examination to detect the recurrence of TGCT early stated that it enhanced their confidence. “*Physical activities,*” “*patience*” and “*accepting physical changes*” helped men experiencing adverse and late events to regain confidence and physically get on with their lives. Survivors who were longer than five years in follow-up care said it was easier to accept this one day than the other.

Sometimes it is a little bit difficult, and you are thinking, hey, I want to go somewhere with my car, and then you have the feeling why won’t it go, and then, you are disappointed again, and that is difficult to accept it. (P4)

A few survivors did not experience aftereffects and said that having cancer could happen to anyone (*“your body is also able to make mistakes”* (P8)). Still, all participants described how regaining confidence in their bodies helped them in various ways to reclaim their lives.

The final phase of the transition consists of finding enduring confidence in one’s own body and associating physical complaints with cancer no longer.

I immediately associated everything that I felt in my body with cancer…the trust was completely gone, and I had to rebuild it from almost nothing … finally it is this trust in my own body that I needed to keep. (P2)

#### Theme: Mentally Being Able to Move On

Nearly all participants saw mental recovery as a large part of their follow-up care, besides the purely physical aspect of being cured of cancer.

You cannot just let someone go after such a drastic event. Not only because of the medical side but also, for some people, because of the mental stuff. I think looking at both sides is a good idea. (P8)

Their transition toward becoming long-term survivors was described as “*personal*” and “*tailored by their needs*.” Follow-up care helped survivors in “*facing*” and “*accepting what happened*.” Half of the participants expressed that their family and friends had an essential role in their mental recovery. Feeling supported and not alone in their mental recovery helped them overcome their cancer experience.

Several participants explained that the moment they realized they no longer needed the checks was the moment they could end follow-up care. For most, mental recovery was not explicitly seen as a part of the hospital treatment. Follow-up care helped them to reach out for specialized psychosocial care outside the hospital. Still, ending follow-up care meant the whole package: full physical and mental recovery from the cancer threat.

If after a while it stops. I think that is quite normal actually because it means you are mentally ready…you have accepted that you no longer need the checks. (P9)

Some survivors, particularly those still in follow-up care after 5 years, continued to feel a catastrophic threat looming over them, although they were medically declared “clean” at the end of follow-up care. They affirmed their anxiety in letting go of the follow-up care and pointed out that follow-up care itself counteracted moving on mentally:

There is a tipping point in what the follow-up is doing. That also triggered me, I did not realize it myself yet, but my environment started to notice it because I still needed it, I thought, that check, it was from mmm, decision activates this erm old very than to remove the future turmoil. I think I should really stop. (P1)

## Discussion and Conclusions

This study gained insight into the perspectives of TGCT survivors’ transition from follow-up care to long-term survivorship. Examining cancer survivorship from the locus of control framework, which uncovered the processes of acceptance and recovery after completion of cancer treatment, is an important addition to the testicular cancer survivorship literature that was previously unexplored. The transition of interviewed TGCT survivors was similar, but individual (life) experiences influenced overcoming a sense of looming doom and strengthening the feeling to get on with life. The core category “*Dealing with back-and-forth forces*” explains the cyclical journey in coping with those feelings to be able or unable to carry on with everyday life. The theoretical model explains the transition’s individuality and circularity in dealing with challenges, which can be used as a guide for providing follow-up care and researching and developing tailored interventions for TGCT survivors. Being aware of the similarities of the transition and the individuality in dealing with the processes might allow healthcare providers to identify and support the stages that TGCT survivors go through toward long-term survivorship.

Previous qualitative studies examining the transition in cancer survivorship confirm the individuality and circularity of the transition and their processes, although age and gender differ (Matheson et al., 2016a; Matheson et al., 2016b; Sherman et al., 2012). In addition, the results of an international Delphi study among oncology nurses on nurses’ perspectives of cancer survivorship mirrored the processes and recalled the transition as an individual journey (Truant et al., 2017). In other Adolescents and Young Adults (AYA) survivorship studies, the need to normalize and move forward with their lives after cancer treatments are similar to the way TGCT survivors wanted to get on with their lives, which is expected since TGCT predominantly affected most men between 18 and 40 years (Matheson et al., 2016a; Smits-Seemann et al., 2017). These qualitative studies suggest that the transition in cancer survivorship is similar for various cancer survivors.

The present, grounded theory suggests that when going through the processes of transition, a TGCT survivor determines not only the speed but also the position of the processes toward long-term survivorship. Speed and position can alternate during the transition due to the individual’s (life) experiences. Survivors who felt difficulties in *“Living beyond the sword of Damocles”* and “*Getting on with one’s life”* demonstrated characteristics of an avoidance-oriented coping style such as not openly talking about their cancer history (not seeking social support) and clinging on to their oncologist, lab results, and imaging after being declared medically clean and at the end of follow-up care (neuroticism) (Taylor & Stanton, 2007). Survivors who learned to regulate their fear of cancer recurrence (control the emotional response to stressors) regained self-confidence and started prioritizing their daily lives over their follow-up care (problem-solving) demonstrated characteristics of approach-oriented coping. Quantitative studies into coping of TGCT survivors demonstrate both the negative effect of avoidance-oriented coping (Rutskij et al., 2010) and the positive effect of approach-oriented coping (Hoyt et al., 2016) in survivorship. These results suggest that promoting an approach-oriented coping style can benefit the transition toward long-term survivorship. In reflection of the internal locus of control, it seemed that survivors who took control over the challenges they encountered in the transition toward long-term survivorship came to a natural end where they realized they no longer needed the checks and could end follow-up care.

### Strengths and Limitations

This study has several strengths. Theoretical saturation was achieved within the 12 interviews and by a diverse and representative sample and affirmed by deviant cases, strengthening methodological consistency. The duration and conditions of the interviews resulted in rich, thick descriptions, further enhancing the study’s credibility. Investigator triangulation and the use of SRQR increased methodological awareness. Self-awareness and sensitivity to the data were achieved by evaluating the interview style, stage-by-stage review by the research team, recording an audit trail, and reflecting on theoretical memos and field notes. A few limitations need to be considered. Despite all efforts to include all sixteen eligible survivors, two survivors refused to participate, and two survivors did not respond to the voicemail. Those refusing did not want to look back at their experiences, yet their perspectives could have been valuable to the study. These survivors could have said something about how they coped with the processes in transition and could add to the theory of the individual pace. It is unknown if not wanting to look back meant moving forward. In addition, the interviews were conducted with two different techniques due to the COVID-19 outbreak. It could influence the methodological consistency, although the interviewer (SW) sent additional information to the web-based interviews to ensure similar interview conditions and studied critical criteria for video interviewing. Moreover, research showed that web-based interviews do not have to affect in-depth interviewing compared to face-to-face interviews (Mirick & Wladkowski, 2019; Vadi et al., 2016).

### Clinical Implications

The findings have implications for clinical practice regarding follow-up care. Firstly, to improve follow-up care, healthcare professionals must understand TGCT survivors’ transition and their ensuing needs toward long-term survivorship. The theoretical model can assist in understanding individual cues that set the motion or alternate the position in the transition. Awareness of how the TGCT survivor copes with the challenges of a “new normal” could uncover those individual cues. Secondly, the study demonstrated the need for tailored interventions in preventing and reducing long-term effects for TGCT survivors because the transition toward long-term survivorship encompasses more than physical recovery. Prior studies report unmet care needs of TGCT survivors regarding the (self-)management of adverse and late events and the availability of community support services (Alexis et al., 2020; Bender et al., 2012; Martin et al., 2013; Shen et al., 2016; Smith et al., 2013). Oncology nursing is well-suited to advance specialized survivorship care due to its holistic view of patient care and coordinating role in healthcare provision (Haylock, 2015). Future research is needed to determine further individual elements that can influence the direction and the speed of the cogwheels within the transition model and how the early determination of personal needs can facilitate the transition.
